# Genome-wide analysis of dysregulated RNA-binding proteins and alternative splicing genes in keloid

**DOI:** 10.3389/fgene.2023.1118999

**Published:** 2023-01-26

**Authors:** Zhen Zhu, Shuangying Ni, Jiali Zhang, Ying Yuan, Yun Bai, Xueli Yin, Zhengwei Zhu

**Affiliations:** ^1^ Hangzhou Plastic Surgery Hospital, Hangzhou, China; ^2^ Department of Dermatology, The First Affiliated Hospital, Institute of Dermatology, Anhui Medical University, Hefei, China; ^3^ The Key Laboratory of Dermatology, Ministry of Education, Anhui Medical University, Hefei, China; ^4^ Department of Plastic Surgery, The First Affiliated Hospital, Anhui Medical University, Hefei, China; ^5^ Functional Experiment Center, School of Basic Medical Sciences, Anhui Medical University, Hefei, China

**Keywords:** keloid, alternative splicing, trauma healing, RNA-bindig proteins, RNA-sequencing (RNA-seq)

## Abstract

**Introduction:** The pathogenesis of keloids remains unclear.

**Methods:** In this study, we analyzed RNA-Seq data (GSE113619) of the local skin tissue of 8 keloid-prone individuals (KPI) and 6 healthy controls (HC) before and 42 days after trauma from the gene expression omnibus (GEO) database. The differential alternative splicing (AS) events associated with trauma healing between KPIs and HCs were identifified, and their functional differences were analyzed by gene ontology (GO) and kyoto encyclopedia of genes and genomes (KEGG) pathways. The co-expression relationship of differentially alternative splicing genes and differentially expressed RNA binding proteins (RBPs) was established subsequently.

**Results:** A total of 674 differential AS events between the KD42 and the KD0 and 378 differential AS events between the HD42 and the HD0 were discovered. Notably, most of the differential genes related to keloids are enriched in actin, microtubule cells, and cortical actin cytoskeletal tissue pathway. We observed a signifificant association between AS genes (EPB41, TPM1, NF2, PARD3) and trauma healing in KPIs and HCs. We also found that the differential expression of healthy controls-specifific trauma healing-related RBPs (TKT, FDPS, SAMHD1) may affect the response of HCs to trauma healing by regulating the AS of downstream trauma healing-related genes such as DCN and DST. In contrast, KPIs also has specifific differential expression of trauma healing related RBPs (S100A9, HspB1, LIMA1, FBL), which may affect the healing response of KPIs to trauma by regulating the AS of downstream trauma healing-related genes such as FN1 and TPM1.

**Discussion:** Our results were innovative in revealing early wound healing-related genes (EPB41, TPM1, NF2, PARD3) in KPI from the perspective of AS regulated by RBPs.

## 1 Introduction

Keloid is a kind of skin fibroproliferative disease, a benign skin tumor formed in the process of wound healing, and there are genetically susceptible individuals (Russell et al., 2010). Keloids are locally aggressive, continually growing, and invade the surrounding normal skin. Surgical removal of keloids has a high recurrence rate, and more severe scars may recur after surgical removal (Shih et al., 2010). Also, individuals with Asian ancestral backgrounds have a high propensity for the development of keloids (Lu et al., 2015). Unfortunately, keloids remain a serious challenge due to the lack of effective treatments. Therefore, there is an urgent need to continue to investigate the mechanisms of keloid development in existing cases to facilitate the elaboration of better treatment plans or early interventions.

Skin tissues from keloid-prone individuals (KPI) were collected and analyzed using genomic and other histological techniques. The study of the molecular phenotype of keloids has revealed the immunological basis and molecular mechanisms of the relevant pathobiological processes, enabling the precise treatment of susceptible individuals and the development of therapeutic drugs targeting specific immune pathways (Jin et al., 2019; Tsai and Ogawa, 2019; Wu et al., 2020; Liu et al., 2022). Therefore, it is necessary to use sequencing technology to explore the molecular mechanism of the occurrence and development of keloids, which will find potential effective targets to guide the prevention and treatment of keloids. Epigenetic mechanisms (including DNA methylation, histone modification, and ncRNA regulation) represent a potential link between the complex interplay of genetics and external risk factors for keloid formation (Lv et al., 2020). SiRNA plays a role in regulating TGF-β-induced polypyrimidine bundle-binding protein in the pathophysiology of keloid.

Alternative splicing (AS) of genes is a major source of protein diversity and is associated with many diseases. AS has been recognized to have an essential function in the development of various cancers but has been poorly studied in keloids. Yan et al. (2015) finds that AS changes in the FGFR2 gene may be related to abnormal epidermis and appendages in keloids. Genome-wide association study had proved rs8032158 SNP is located in the intron between exons 6 and 7 of the NEDD4 gene is associated with keloid in Japanese population (Nakashima et al., 2010). It is speculated that NEDD4 intron SNP may affect the AS of NEDD4 mRNA in exons 6 and 7, which may be related to the inflammation development during keloid formation (Fujita et al., 2019). Fibronectin has various functions that are effectuated by different protein isoforms (including EDA and EDB) generated through AS of genes (Hynes, 1986; Moretti et al., 2007). Knockdown of EDB inhibits TGF-1-induced cell proliferation and suppresses collagen deposition in keloid fibroblasts (Cui et al., 2020). However, no studies comprehensively identify and explore the role of AS and its regulatory mechanism in keloid tissues.

The loss of function or abnormal expression of RNA-binding proteins (RBP) will affect the AS changes and then affect the proliferation and metastasis of cancer cells. The current study on keloid found that RBP LARP6 can regulate collagen synthesis in fibroblasts and promote the proliferation and invasion of tumor cells, thus enabling the rapid growth and invasion of fibroblasts in keloid lesions (Chen et al., 2022). RBP FXR1 can also directly bind to circRNAs, thereby reducing fibroblast proliferation and extracellular matrix accumulation and increasing the level of apoptosis (Wang et al., 2021). However, the mechanism of RBP as a regulator of AS gene in keloid remains unclear. Therefore, we hope to further identify functionally abnormal RBPs in keloid and explore their correlation with abnormal AS during keloid trauma healing, thereby revealing the mechanisms leading to keloid formation and suggesting potential targets for the treatment or prevention of keloid. In our study, we downloaded RNA-seq data of skin tissues including healthy controls (HC) and KPI from GSE113619 at 0 days before surgery and 42 days after surgery. We further analyzed the differential AS events between HC and KPI to establish the regulatory network involved in differential splicing genes. These AS events will help to predict trauma healing in keloid patients, and to reveal the potential mechanism of splicing genes.

## 2 Materials and methods

### 2.1 Retrieval and process of public data

Searching in the GEO database with “keloid” as a keyword, public sequence data files GSE113619 (Onoufriadis et al., 2018) were downloaded from the Sequence Read Archive (SRA). SRA Run files were converted to fastq format with NCBI SRA Tool fastq-dump. The raw reads were trimmed of low-quality bases using a FASTX-Toolkit (v.0.0.13; http://hannonlab.cshl.edu/fastx_toolkit/). Then the clean reads were evaluated using FastQC (http://www.bioinformatics.babraham.ac.uk/projects/fastqc).

### 2.2 Reads alignment and differentially expressed gene analysis

Clean reads were aligned to the GRCh38 genome by HISAT2 (Kim et al., 2019). Uniquely mapped reads were ultimately used to calculate the read number and fragments per kilobase of exon per million fragments mapped (FPKM) for each gene. The expression levels of genes were evaluated using FPKM. When we do gene differential expression analysis, we choose the software DEseq2 (Love et al., 2014) and use the volcano plot for visualization. DEseq2 will model the original reads and use the scale factor to explain the difference in Library depth. Then, DEseq2 estimates the gene dispersion and reduces these estimates to generate more accurate dispersion estimates, to model the read count. Finally, the model of the negative binomial distribution is fitted by DEseq2, and the hypothesis is tested by the Wald test or likelihood ratio test. DEseq2 can be used to analyze the differential expression between two or more samples, and the analysis results can be used to determine whether a gene is differentially expressed by fold change (FC) and false discovery rate (FDR).

**There are two important parameters**

1) FC: Fold change, the absolute ratio of expression change.
2) FDR: False discovery rate.

**The criteria of significant differential expression were as follows**

FC ≥ 2 (up) or ≤0.5 (down), FDR ≤0.05.

### 2.3 Principal component analysis plot

Principal component analysis (PCA) of different groups’ expression profiles was performed using the R package “factoextra”.

### 2.4 Identification of differentially expressed RBPs

The software DEseq2, which is specifically used to analyze the differentially expressed genes (DEG), was applied to screen the raw count data for DEGs. The results were analyzed based on the fold change (FC ≥ 2 or ≤1/2) and false discovery rate (FDR ≤0.05) to determine whether a gene was differentially expressed. The expression profile of differentially expressed RBPs was filtered out from all DEGs according to a catalog of 2,141 RNA-binding proteins (RBPs) retrieved from two previous reports (Castello et al., 2012; Gerstberger et al., 2014; Castello et al., 2016; Hentze et al., 2018).

### 2.5 Alternative splicing analysis

Regulatory alternative splicing events (RAS) among different groups were defined and quantified using the SUVA pipeline (Cheng et al., 2021). The different splicing of each group was analyzed. Reads proportion of SUVA AS event (pSAR) of each AS event was calculated. In the working principle of SUVA, five different types of AS event models are defined based on splice site usage changes. One splice site was shared and the other was alternative (alt3p, alt5p). Both splice sites are optional (olp, contains); two front splice sites are used, or neither is used (ir).

The alternative splicing events (ASEs) and regulated alternative splicing events (RASEs) between the samples were defined and quantified by using the ABLas pipeline as described previously Xia et al. (2017). CELF1 preferentially binds to exon-intron boundary and regulates alternative splicing in HeLa cells. Biochim Biophys Acta 1860. Jin et al. (2019). Transcriptome analysis reveals the complexity of alternative splicing regulation in the fungus Verticillium dahliae. Bmc Genomics 18, 130. In brief, ABLas detection of ten types of ASEs was based on the splice junction reads, including exon skipping (ES), alternative 5′splice site (A5SS), alternative 3’splice site (A3SS), intron retention (IR), mutually exclusive exons (MXE), mutually exclusive 5′UTRs (5pMXE), mutually exclusive 3′UTRs (3pMXE), cassette exon, A3SS&ES, and A5SS&ES. For sample pair comparison, Fisher’s exact test was selected to determine statistical significance, using the alternative reads and model reads of the samples as input data. We calculated the changed ratio of alternatively spliced reads and constitutively spliced reads between compared samples, which was defined as the RASE ratio. The RASE ratio ≥0.2 and *p*-value ≤0.05 were set as the threshold for RASEs detection. For repetition comparison, Student’s t-test was performed to evaluate the significance of the ratio alteration of AS events. Those events which were significant at a *p*-value cutoff of 0.05 were considered non-intron retention (NIR) RASEs.

### 2.6 Co-expression analysis

Co-expression analysis was performed for all differentially expressed RBP and RAS (pSAR≥50%). Meanwhile, the Pearson correlation coefficient between differentially expressed RBP and RAS was calculated, and DERBP-RAS relationship pairs satisfying the absolute value of correlation coefficient ≥0.6 and *p*-value ≤0.01 were screened.

### 2.7 Functional enrichment analysis

To sort out functional categories of DEGs, Gene Ontology (GO) terms and KEGG pathways were identified using KOBAS 2.0 (Xie et al., 2011). Hypergeometric test and Benjamini–Hochberg FDR controlling procedure were used to define the enrichment of each term.

## 3 Results

### 3.1 Analysis of differentially expressed genes

**FIGURE 1:**
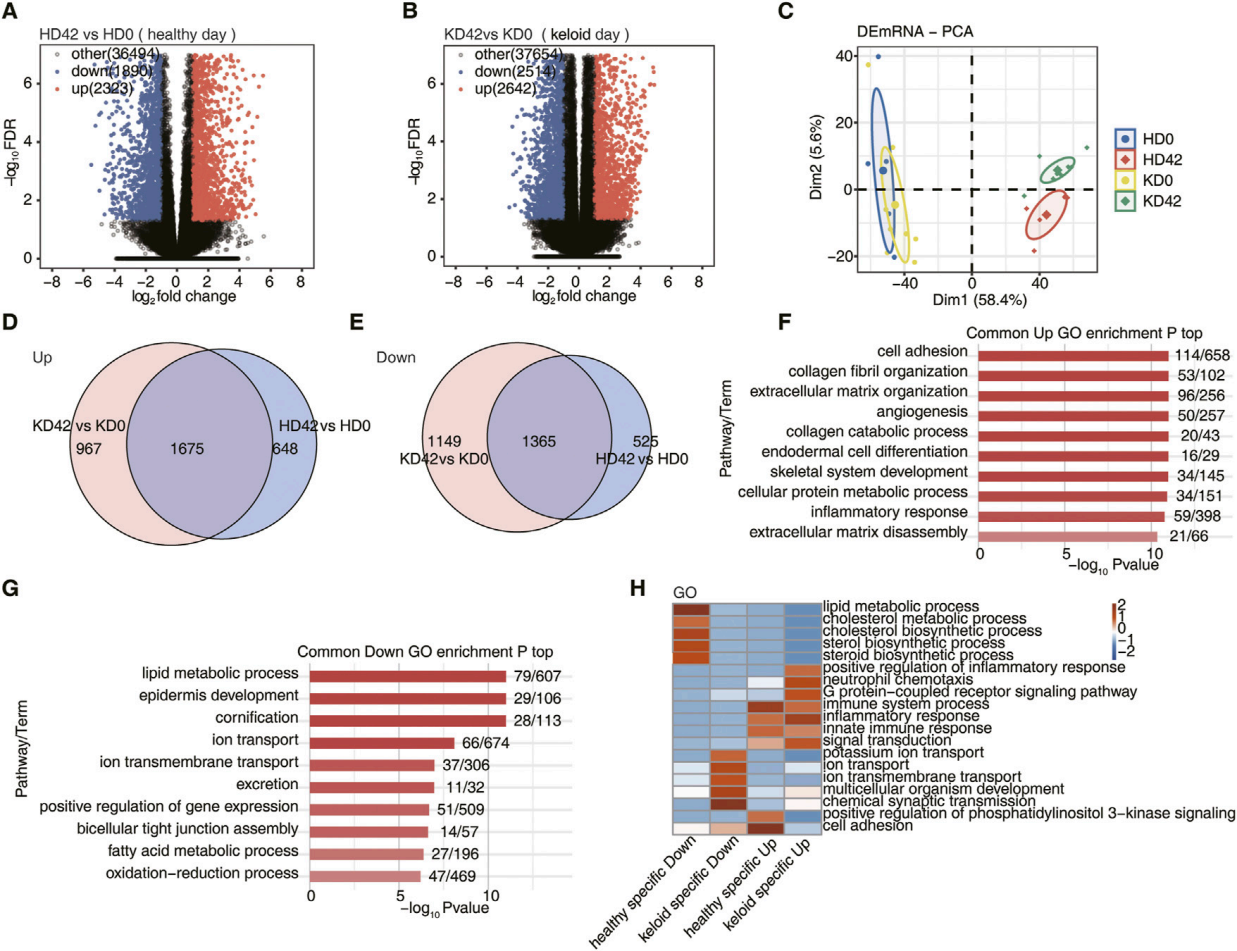
Differential gene analysis of local skin repair in patients with KPI and HC after 42 days of a wound. **(A)** Volcano plots to present all DEGs between HD42 (healthy day) and HD0 samples with DESeq. FDR ≤0.05 and FC (fold change) ≥ 2 or ≤0.5. **(B)** Volcano plots to present all DEGs between KD42 (keloid day) and KD0 samples with DESeq. FDR ≤0.05 and FC (fold change) ≥ 2 or ≤0.5. **(C)** Principal component analysis (PCA) based on FPKM value of all DEG. The ellipse for each group is the confidence ellipse. **(D)** Venn diagram showing the up-regulation overlap between KPI and HC. **(E)** Venn diagram showing the downregulation overlap between KPI and HC. **(F)** Bar plot showing the most enriched GO biological process results of the Common up-regulation. **(G)** Bar plot showing the most enriched GO biological process results of the Common down-regulation. **(H)** The top 5 most enriched GO terms (biological process) were illustrated for specific genes among the different groups. The color scale shows the row-scaled significance (-log10 corrected *p*-value) of the terms.

In transcriptomic data based on skin tissue samples from 8 KPIs and 8 healthy controls (HC) before and 42 days after surgery, there were 2,323 up-regulated differentially expressed genes (DEGs) and 1890 down-regulated DEGs between the HD42 (healthy day) and HD0 (Figure 1A). However, 2,642 genes were up-regulated, and 2,514 genes were down-regulated between the KD42 (keloid day) and KD0 (Figure 1B). This suggests that both KPI and HC cause local genetic changes after 42 days of trauma to the skin. Next, we will study in depth the skin barrier recovery of KPI and HC 42 days after skin trauma.

The PCA showed that HD0 and KD0 were fused, indicating that the transcriptomic genes of HC and KPI were very similar before the trauma. However, the KD42 was significantly separated from the HD0 and KD0. It was also substantially different from the HD42 indicating that the local skin transcription level of KPI was significantly changed after 42 days of trauma. It was also considerably different from the local skin repair of HC after 42 days of trauma (Figure 1C). To investigate the mechanisms responsible for the significant differences in KD42, we next performed a further analysis of the repair mechanisms underlying the local differences between KPI and HC-traumatized skin. Next, we compared the upregulated and downregulated DEGs of KPI and HC, respectively. We found 648 up-regulated DEGs and 525 down-regulated DEGs in HC. Upregulation of 967 DEGs and downregulation of 1,149 DEGs specific to KPI. Up-regulation of 1,675 genes and down-regulation of 1,365 genes co-occurred between KPI and HC groups (Figures 1D, E). We found significantly more specific DEGs for KPI compared to HC, suggesting that after skin trauma, skin repair genes for KPI undergo unexpected changes after skin injury.

To understand the function of DEGs, we conducted a GO analysis on common genes of local skin wound repair in KPI and HC after 42 days of trauma. We found that during the repair process of two kinds of local skin wounds, most up-regulated DEGs were enriched in biological pathways such as cell adhesion, collagen fiber organization, extracellular matrix organization, angiogenesis, collagen catabolic process, endodermal cell differentiation, cellular protein metabolism process, inflammatory reaction and extracellular matrix disintegration (Figure 1F). These pathways all seem to be involved in cell repair after trauma. Most down-regulated genes were enriched in biological pathways such as lipid metabolic process, epidermis development, cornification, ion transport, ion transmembrane transport, excretion, positive regulation of gene expression, bicellular tight junction assembly, fatty acid metabolic process, and oxidation-reduction process (Figure 1G). These down-regulated pathways are more likely to provide an environment for epidermal skin repair.

We then analyzed the specific DEGs of both KPI and HC to determine how their skin repair mechanisms differ. First, GO analysis of specific DEGs 42 days after trauma in KPI and HC. To visualize the differences between HC and KPI, all DEGs enriched in the top five biological pathways were subjected to class clustering analysis. Finally, we found that specific up-regulated DEGs in HC were mainly related to cell adhesion and immune system process biological pathways. Specific down-regulated DEGs in HC were primarily associated with biological pathways of the lipid metabolic process. We also found that KPI, specifically up-regulated DEGs, were mainly related to positive regulation of inflammatory responses, neutrophil chemotaxis, immune system process, inflammatory response, innate immune response, and other immune inflammation, with significant enrichment in biological pathways of inflammatory response. Specific down-regulated DEGs of KPI were related to the transport functions of potassium ion transport, ion transport, ion transmembrane transport, chemical synaptic transmission, and other cell membrane substances, among which the biological pathway of chemical synaptic transmission was the most obvious (Figure 1H). We found that most up-regulated DEGs in HC and KPI were enriched in immune-inflammatory pathways. Still, the cell adhesion function in HC was more robust than in keloids, indicating that healthy people promote more excellent skin repair.

### 3.2 Analysis of alternative splicing

Previous studies have shown that the epithelial-mesenchymal transition in keloids is accompanied by abnormal alternative splicing of FGFR2 IIIb/IIIc (Yan et al., 2015). In addition to collagen overexpression in keloid lesions, the expression of the fibronectin splice variant cFN-EDA was significantly upregulated in keloid tissues, affecting wound healing (Andrews et al., 2015). We, therefore, speculate that genetic AS events or AS regulators may influence the development of keloid.

**FIGURE 2:**
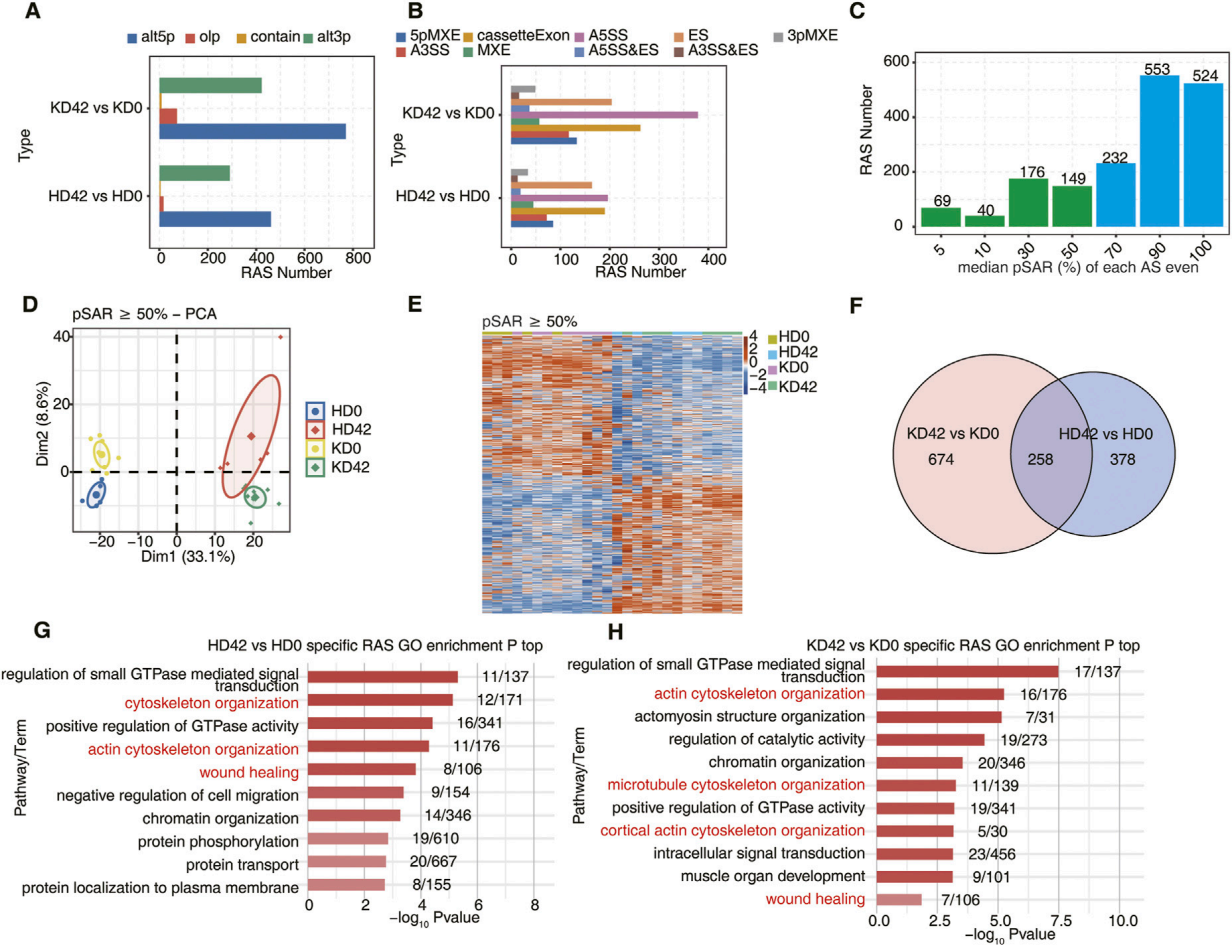
Analysis of alternative splicing during local skin repair in KPI and HC after 42 days of a wound. **(A)** Barplot showing the number of regulatory AS detected by SUVA in each group. ‘alt3p’ indicates the model that 5ʹ splice site is shared and 3ʹ splice site is alternative. “alt5p” indicates the model that 3ʹ splice site is shared and 5ʹ splice site is alternative. “olp” indicates a model that both splice sites are different but part of the splice junction are overlapped. “Contain” indicates a model that both splice sites are different but one splice junction is contained in another splice junction. **(B)** Splice junction constituting RAS events detected by SUVA was annotated to classical AS event types. And the number of each classical AS event type was shown with a barplot. **(C)** Barplot showing RAS with different pSAR. RAS which pSAR (Reads proportion of SUVA AS event) ≥ 50% were labeled. **(D)** Principal component analysis (PCA) based on RAS of pSAR ≥50%. The ellipse for each group is the confidence ellipse. **(E)** The Heatmap showing the splicing ratio of RAS (PSAR ≥50%). **(F)** Venn diagram showing overlap of RAS id between KPI (day42 and day0) and HC (day42 and day0) groups. **(G)** Bar plot showing the most enriched GO biological process results for specific RAS (pSAR ≥50%) in the HC group. **(H)** Bar plot showing the most enriched GO biological process results for specific RAS (pSAR ≥50%) in the KPI group.

For the analysis of transcript levels in HC and KPI, we still do not know the mechanism of the difference, so we conducted an in-depth study of the post-transcriptional levels of DEGs. First, we used the recently published AS analysis method, Splice sites Usage Variation Analysis (SUVA), to analyze and identify regulatory AS (RAS) events based on splice sites. Most of the current AS analysis tools are powerless to analyze complex splicing, and SUVA decomposes complex splicing events into five types of splice junction pairs. By analyzing real and simulated data, SUVA showed higher sensitivity and accuracy in detecting AS events than the compared methods (Cheng et al., 2021). The difference in the proportion of reads used for the same splice event between the two sample groups was compared, with a significant difference RAS event screening criterion of *p*-value ≤0.05, identifying the type of RAS event (Figure 2A). We found that there were different RAS event genes between KPI and HC after trauma, and alt5p and alt3p were the main types of RAS events. However, the number of alt5p and alt3p types between KD42 and KD0 was more than between HD42 and HD0. The analysis of classical AS events showed that many AS events, such as A5SS, cassetteExon, and ES, could be identified between KD 42 and KD 0 and between HD42 and HD0 groups. In addition, the number of AS types in keloids was more than that in healthy people. We speculated that AS event genes played an essential role in the local skin repair mechanism after keloid trauma (Figure 2B).

During the study, many RAS genes accounting for more than 50% of pSAR (proportion of Reads to SUVA AS events) were found (Figure 2C). Next, principal component analysis (PCA) was performed based on the pSAR values of the differential RAS events in the samples. We discovered that KD42 was significantly separated from KD0, and HD42 was also considerably separated from HD 0. Notably, KD0 was more significantly separated from HD0, indicating that the post-transcriptional AS event genes significantly differed in HC and KPI. These results suggest an important pathogenic role of AS event genes between HC and KPI (Figure 2D). Cluster analysis of the samples was performed based on the pSAR values of these differential RAS events in each sample. Genes for differential RAS events were similar between KD0 and HD0, and genes for differential RAS events were also identical between KD42 and HD42. However, there were significant differences between HC and KPI before and after trauma (Figure 2E).

Using Venn plot analysis, we found 674 specific RAS events between the KD42 and KD0 and 378 specific RAS events between the HD42 and HD0 (Figure 2F). However, there was little difference in the number of different RAS events between KPI and HC (Supplementary Figure S1A). The number of splicing event genes in KPI was significantly more than that in HC, indicating the importance of RAS event genes in KPI in wound repair. We also found that the genes with specific differential RAS events between HD42 and HD0 were mainly related to cytoskeletal organization, actin cytoskeletal organization, and wound healing (Figure 2G). The genes with specific differential RAS events between KD42 and KD0 were mainly related to actin cytoskeleton organization, microtubule cytoskeleton organization, cortical actin cytoskeleton organization, and wound healing (Figure 2H). Moreover, the KD0 group had genes with specific expression of RAS events enriched in the regulation of cell cycle and actin cytoskeleton organization compared to HD0 (Supplementary Figure S1B). The KD42 group had more genes with specific expression of RAS events enriched in biological pathways such as cell-matrix adhesion (Supplementary Figure S1C). These results suggest a significant alteration of specific differential AS events in KPI compared to HC, which we speculate may play a role by influencing the biological behavior of keloid fibroblasts during wound healing.

### 3.3 Individual variance analysis of AS

**FIGURE 3:**
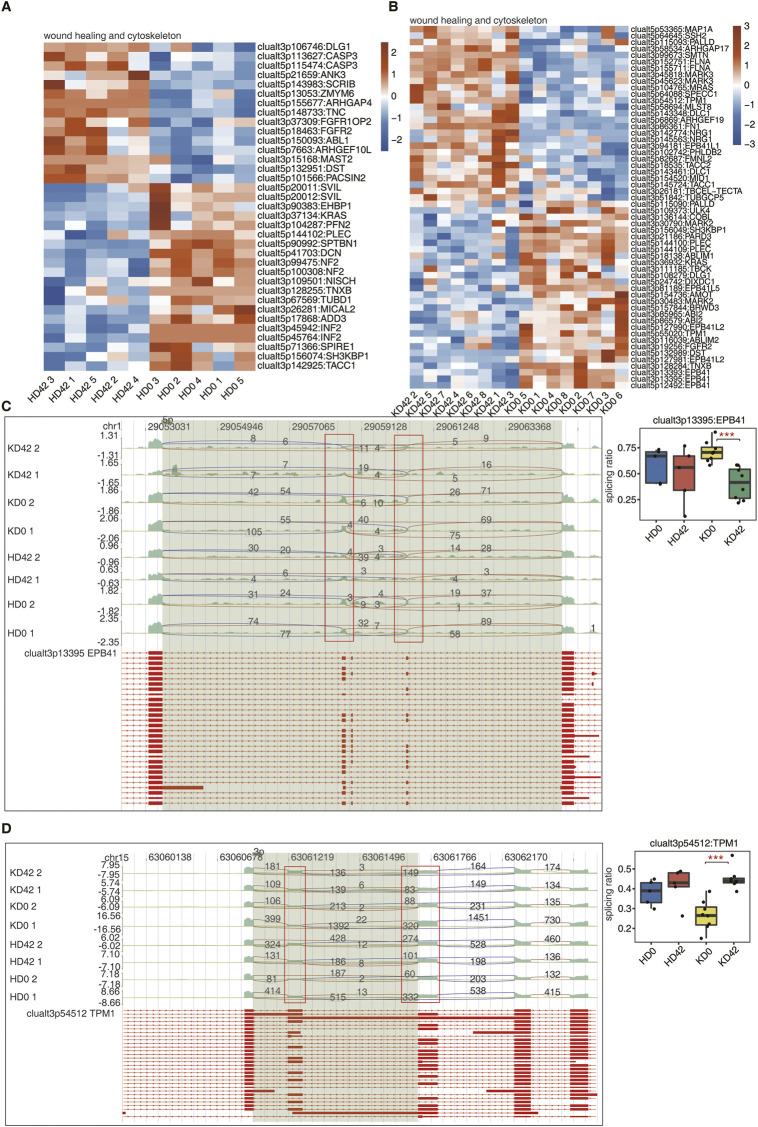
Analysis of differential AS of wound healing-associated genes in skin tissue from KPI and HC. **(A, B)** The Heatmap diagram shows the splicing ratio of wound healing and cytoskeleton in the KPI and HC. **(C)** Visualization of junction reads distribution of erythrocyte membrane protein band 4.1 (EBP41) in AS events clualt3p13395 from different groups. Splice junctions were labeled with SJ reads the number, and the altered exon was marked out with a red box. The splicing events model is shown in the top panel. Boxplot in the bottom panel showing the splicing ratio profile of the splicing event from EBP41. Boxplot showing splicing ratio of clualt3p13395 EPB41 on the right. *p ≤ 0.05, **p ≤ 0.01,***p ≤ 0.001. **(D)** Visualization of junction reads distribution of encoding tropomyosin (TPM1) in AS events clualt3p54512 from different groups. Splice junctions were labeled with SJ reads the number, and the altered exon was marked out with a red box. The splicing events model is shown in the top panel. Boxplot in the bottom panel showing the splicing ratio profile of the splicing event from EBP41. Boxplot showing splicing ratio of clualt3p54512 TPM1 on the right. *p ≤ 0.05, **p ≤ 0.01,***p ≤ 0.001.

These results indicate that we should focus on trauma healing-related AS genes in KPI during the trauma repair process. To further explore the differences in trauma healing patterns between KPI and HC, we extracted all trauma healing-associated differential AS events that occurred on genes in the Figure 2 red-labeled pathway. Sample clustering analysis was performed based on the pSAR values of these AS events. Trauma healing-related AS genes of HD42 were separated from HD0, indicating significant differences in trauma healing genes. KPI 0 days and 42 days were also clearly separated, indicating that trauma healing genes also differed significantly (Figures 3A, B).

EPB41, TPM1, NF2, and PARD3, these four genes with differentially expressed AS events were different among the four groups of samples. The reads distribution of the above four differentially expressed AS events that occurred on genes related to wound healing is shown (Figures 3C, D; Supplementary Figure S2A–C). Among them, the splicing ratio of AS events that occurred on EPB41 and PARD3 were significantly decreased and the splicing ratio of AS events that occurred in TPM1 was significantly increased in KD42. And two AS events, alt3p13395 and alt5p12492, occurred in EBP41. This suggests that EBP41, PARD3, and TPM1 play important roles in the early stages of KPI wound healing and may impede physiological wound healing progression.

### 3.4 Co-expression analysis of specific RBPs and ASs

**FIGURE 4:**
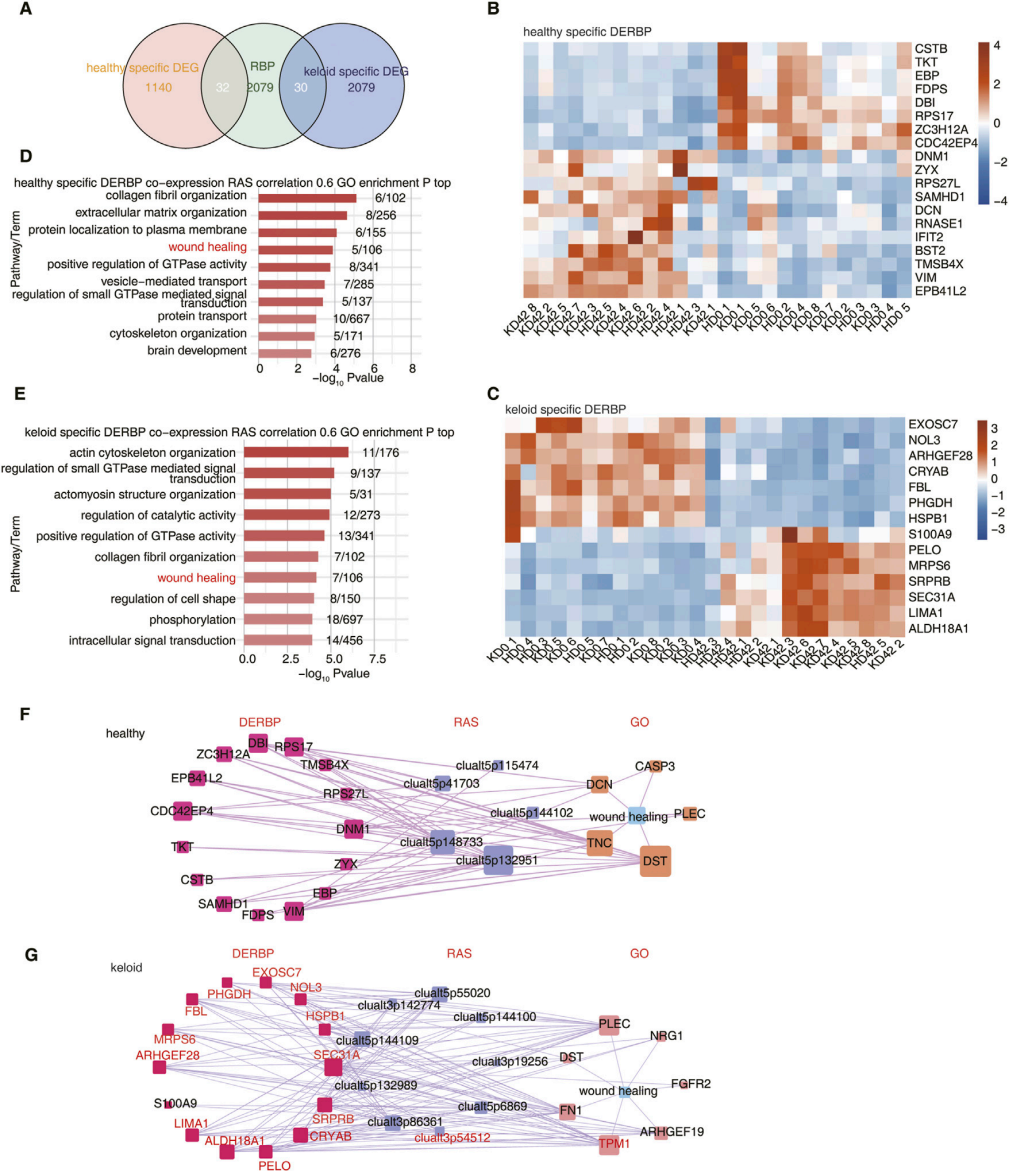
Differentially expressed RBPs potentially affect AS of wound healing-associated genes in KPI and HC. **(A)** Venn diagram showing the overlap of DEG and RBP. **(B)** The Heatmap showing the expression profile of HC-specific DERBP. **(C)** The Heatmap showing the expression profile of KPI-specific DERBP.**(D)** Bar plot showing the most enriched GO biological process results of RAS co-expressed by specific DERBP in the HC group. Cutoffs of *p* ≤ 0.01 and Pearson coefficient ≥0.6 or ≤ −0.6 were applied to identify the co-expression pairs. **(E)** Bar plot showing the most enriched GO biological process results of RAS co-expressed by specific DERBP in the KPI group. Cutoffs of *p* ≤ 0.01 and Pearson coefficient ≥0.6 or ≤ −0.6 were applied to identify the co-expression pairs. **(F)** Network diagram showing the wound healing pathway of RAS co-expressed by specific DERBP in HC. **(G)** Network diagram showing the wound healing pathway of RAS co-expressed by specific DERBP in KPI. The red marked DERBP stands for Pearson coefficient ≥0.6.

RNA-binding proteins (RBPs) are a class of highly conserved proteins that play pivotal roles in modulating gene expression at the post-transcriptional level (Van Nostrand et al., 2020). RBPs can regulate gene expression through post-transcriptionally influencing all manner of RNA biology, including alternative AS (Hu et al., 2020). The differential expression of RBPs (DERBPs) between KPI and HC may lead to abnormal AS in skin tissues. The specific DEGs of KPI and HC were intersected with the reported potential human RBPs genes, in which HC had 32 specific DERBPs and KPI had 30 specific DERBPs (Figure 4A). These DERBPs may be involved in the regulation of differential splicing events genes in KPI and HC. We found that 19 DERBPs were abnormally up-or down-regulated in HC, which may affect and regulate the AS of downstream genes (Figure 4B). In contrast, for the KPI, 14 DERBPs were distributed significantly differently in KD 42 than in the other three groups. We speculate that these DERBPs may influence and regulate AS of more keloid post-traumatic healing-related downstream genes (Figure 4C).

To further explore the regulatory relationship between DERBPs and differential AS events, co-expression analysis was performed using specific DERBP genes and pSAR values of specific trauma healing-associated differential AS events between HC and KPI. We identified differential AS events co-expressed by these typical DERBPs genes, extracted the genes where the co-expressed differential splicing events were located, and performed GO functional analysis. It could be seen that the co-expressed genes were mainly enriched in the trauma healing-related pathway (Figures 4D, E) and were also enriched in extracellular matrix-related pathways such as focal adhesion and ECM-receptor interaction (Supplementary Figure S3A–B). We further extracted the differential AS events of wound healing-related genes and the co-expressed DERBPs and constructed the co-expression network of KPI and HC respectively. It was found that the local skin wound healing-related genes in HC included TKT, FDPS, SAMHD1, and other abnormally expressed DERBPs. They may regulate the AS of downstream wound healing-related genes such as DCN and DST (Figure 4F; Supplementary Figure S3C). The trauma healing-related genes of KPI include abnormally expressed DERBPs such as S100A9, HspB1, LIMA1, and FBL, which may regulate the AS of downstream trauma healing-related genes such as FN1 and TPM1(Figure 4G; Supplementary Figure S3D).

## 4 Discussion

In this study, we analyzed the alternative splicing pattern of genes in the local skin tissue of keloid susceptible individuals (KPI) and healthy controls (HC) before and 42 days after trauma and identified four differential AS genes, EPB41, TPM1, NF2, and PARD3, associated with trauma healing between KPI and HC. We also established the co-expression relationship between differential AS genes, and differentially expressed RBPs. We found that the differential expression of RBPs including TKT, FDPS, SAMHD1, and other trauma healing-related genes specific to healthy individuals may affect the healing response to trauma in healthy individuals by regulating the AS of downstream trauma healing-related genes such as DCN and DST. In contrast, KPI also has specific trauma healing-related genes, including the differential expression of RBPs such as S100A9, HSPB1, LIMA1, and FBL, which may affect the response of KPI to trauma healing by regulating the AS of downstream trauma healing-related genes such as FN1 and TPM1. Our results preliminarily reveal the mechanism of AS affects KPI trauma healing and keloid formation, and provide potential targets for the treatment or prevention of keloid.

Nardini et al. (2016) finds that during wound healing, cell adhesion promotes sustained migration as cells pull neighboring cells into the wound. Lipid metabolism ensures the synthesis of structural lipids required for the epidermal barrier and lipids secreted and covering the skin and fur (Kruse et al., 2017). The literature above indicates that cell adhesion effectively promotes wound healing in normal human skin repair processes. When the integrity of the skin barrier is impaired, the synthesis of structural lipids required by the skin and the secretion of lipids will be affected. The mechanism of keloid formation may be related to the continuous up-regulation of proinflammatory gene expression in keloid lesions (Dong et al., 2013). In addition, researchers found that mast cells and Langerhans cells are present in extremely high numbers in keloids, and that mast cells secrete interleukins and growth factors that stimulate collagen production and promote the scarring process (Wilgus et al., 2020). Persistent activation of inflammatory pathways leads to prolonged hyperactivation of fibroblasts and myofibroblasts, which is necessary for excessive collagen formation in abnormal scarring (Zhang et al., 2020). Our results are consistent with previous studies, but we are not aware of any studies focusing on the effects of post-transcriptional regulation on keloids. So, our study provides a new perspective for the pathogenesis of keloid.

Moreover, AS of the FGFR2 gene switched the predominantly expressed isoform from FGFR2-IIIb to -IIIc, concomitant with the decreased expression of ΔNp63 and TAp63, which changes might partially account for abnormal epidermis and appendages in keloids (Yan et al., 2015). Wound healing is a complex biological process that includes inflammation, migration, differentiation, and proliferation of fibroblasts, endothelial cells and keratinocytes, neointima formation, and synthesis and degradation of extracellular matrix. Changes in cytoskeletal dynamics may affect single-cell repair (Boucher and Mandato, 2015). The cytoskeletal network drives and organizes cell movements to promote rapid repair (Rothenberg and Fernandez-Gonzalez, 2019). These suggests that the cytoskeleton promotes wound repair. Coincidentally, the genes specific to KPI are enriched in functional biological pathways similar to those of HC. However, more genes in KPI were enriched in actin, microtubule cells, and cortical actin cytoskeleton pathway. We suggest that possibly post-traumatic healing is stronger in KPI than in HC during cytoskeletal remodeling, again implying that AS event genes play an essential role in KPI.

EPB41, erythrocyte membrane protein band 4.1, which is encoding protein 4.1R. First identified and abundantly expressed in human erythrocytes, protein 4.1R (4.1R) represents a member of the protein 4.1 family that plays a crucial role in maintaining the structural stability of erythrocytes (Parra et al., 1998) (Draberova et al., 2019). Protein 4.1R acts as an adaptor, linking membrane proteins to the cytoskeleton, and is involved in many cell events such as cell activation and differentiation, and cytokine secretion (Liang et al., 2020). We believe that the decreased expression of EPB41 in keloid-susceptible patients is related to the increase of inflammatory cytokines in early wound healing and the inability to progress to the proliferative stage of healing.

The partition-deficient gene PARD3 encodes Pard3, a scaffolding protein and a member of the Par protein family. The Par complex is a polar complex that maintains cell polarity and is essential for intercellular adhesion, tight junction formation, and intercellular signaling (Li et al., 2017). The role of Pard3 is particularly important in the Par complex, and its abnormal expression will lead to loss of cell polarity and even tumor tumorigenesis (Wen and Zhang, 2018). Existing studies have concluded that Keloid recurrence and invasiveness may involve aberrant genetic activation of epithelial-mesenchymal transition during the abnormal wound-healing process (Hahn et al., 2016) (Yan et al., 2015) (Yuan et al., 2019). We believe that the downregulation of PARD3 expression in KD42 may lead to a decrease in keratinocyte polarity, which in turn exacerbates the aggressiveness of the keloid.

Tpm, encoding tropomyosin, is one of the major protein partners of actin. Tpm molecules are α-helical coiled-coil protein dimers forming a continuous head-to-tail polymer along the actin filament. Human cells produce a large number of Tpm isoforms that are thought to play a significant role in determining actin cytoskeletal functions (Marchenko et al., 2021). Systemic TPM1 was an immune-related molecule that elicited inflammation by phosphorylating PKA and regulating FABP5/NF-κB signaling (Li et al., 2022). MiR-29c seems to exert an inhibitory effect on myofibroblast activation, such as collagen gel contractility and migration ability, *via* suppressing TPM1 (Yang et al., 2022). The TPM1 promotes cell movement during wound healing (Lees et al., 2013). We suggest that upregulation of the alternative splicing gene TPM1 in KPI may have a great effect on the local inflammatory response and excessive migration and proliferation of fibroblasts in keloid, and may be a potential therapeutic target for keloid.

In HC with abnormal expression of RBP, TKT blockade directly or indirectly reduces proinflammatory and angiogenic cytokine production by M1 macrophages by reducing migration and proliferation of endothelial cells, thereby reducing angiogenesis and inflammation *in vitro* (Parma et al., 2020). RPS17 mutation leads to impaired translation, which may cause Diamond-Blackfan anemia (Cmejla et al., 2007). DCN affects stromal fibrogenesis and corneal healing (Gupta et al., 2022) and may delay skin healing (Jarvelainen et al., 2006). DST mutations cause the skin phenotype of epidermolysis bullosa simplex (Cappuccio et al., 2017). These genes work together to allow the wound-healing process to proceed steadily and without overgrowth in healthy individuals. And in KPI with abnormal expression of RBP, overexpression of S100A9 has a proinflammatory effect and damages macrophage differentiation, and may even expand skin inflammation and delay skin repair (Franz et al., 2022). HspB1 may increase the number of small fibers in mice and cause delayed muscle growth (Kammoun et al., 2016). Lima1 is enriched on cortical actin filaments and can stabilize the cortex to inhibit membrane blister formation, and its endogenous downregulation leads to membrane blebbing and apoptotic responses (Duethorn et al., 2022). The FN1 promotes collagen synthesis (Poor et al., 2022).

Although there are still some limitations in our study, such as too small a sample size and lack of clinical validation. Functional verification will be carried out in the following study. However, our results are innovative in revealing early wound healing-related genes (EPB41, TPM1, NF2, PARD3) in keloid patients from the perspective of alternative splicing regulated by RBP. We also identified a group of RBPs (S100A9, HspB1) and their regulated AS genes (FN1, TPM1) that are aberrantly expressed in keloid, providing a new clue for the treatment or prevention of keloid.

## Data Availability

The original contributions presented in the study are included in the article/Supplementary Material, further inquiries can be directed to the corresponding author.
