# Supplementary material for: Genome-wide analysis of dysregulated RNA-binding proteins and alternative splicing genes in keloid
Source: Front Genet. 2023 Jan 26;14:1118999. doi: 10.3389/fgene.2023.1118999 (PMC9908963; doi:10.3389/fgene.2023.1118999)
Supplement: Supplementary file 1 [file DataSheet1.docx]

Supplementary Material

Genome-wide Analysis of Dysregulated RNA-Binding Proteins and Alternative Splicing Genes in Keloid

Zhen Zhu^1†^, Shuangying Ni^2,3†^, Jiali Zhang^2,3†^, Ying Yuan^2,3†^, Yun Bai^4^, Xueli Yin^5^, Zhengwei Zhu^2,3*^

*** Correspondence:** Zhengwei Zhu: [ahmuzzw@163.com](mailto:ahmuzzw@163.com)


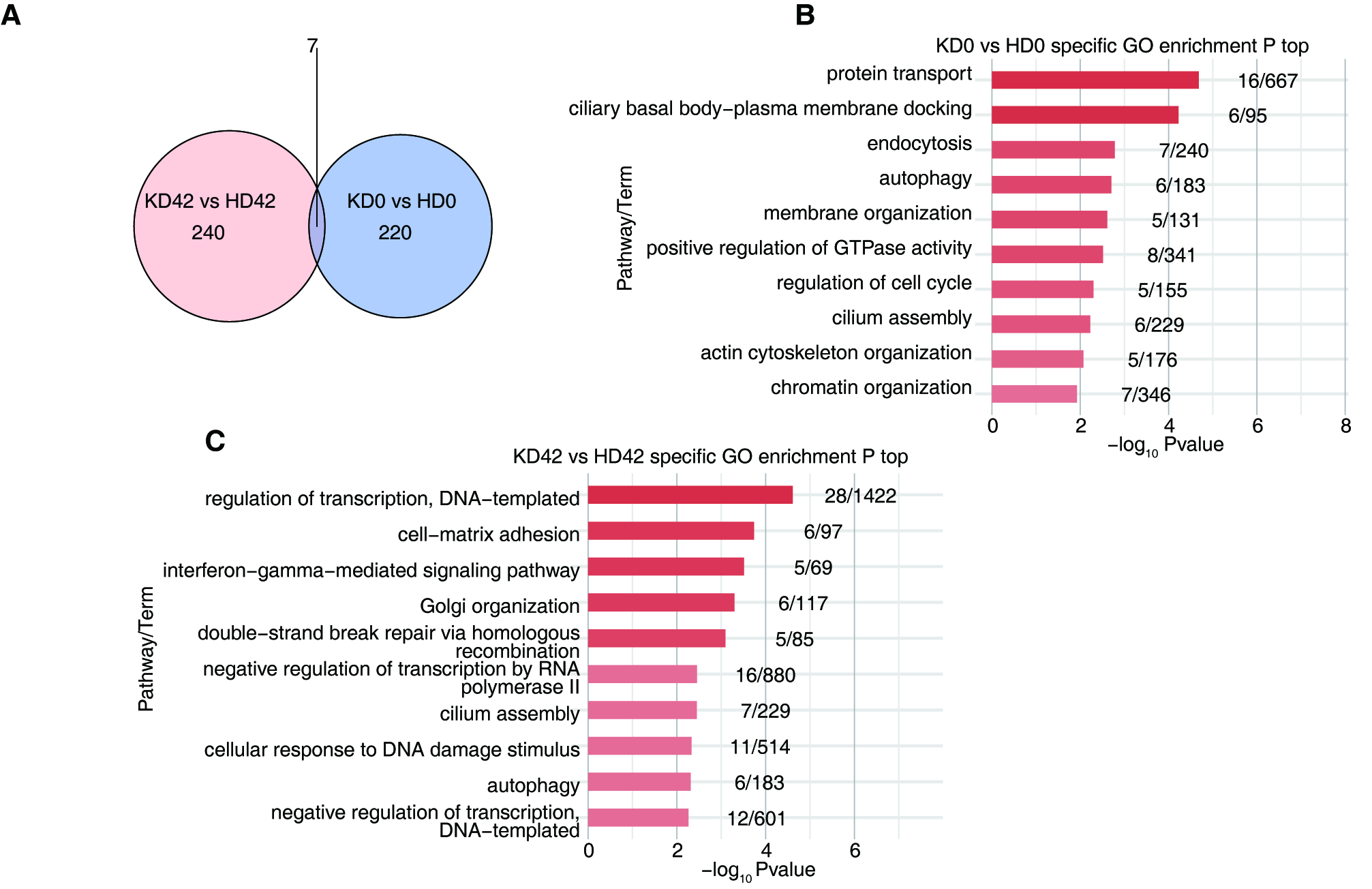


**Supplementary Figure 1:**Analysis of alternative splicing during local skin repair in KPI and HC after 42 days of a wound. (A) Venn diagram showing overlap of RAS id between KD 42 and HD 42 and KD0 and HD0 groups. (B) Bar plot showing the most enriched GO biological process results for specific RAS(pSAR ≥ 50%)in the KD0 and HD0. (C) Bar plot showing the most enriched GO biological process results for specific RAS(pSAR ≥ 50%) in the KD42 and HD42 groups.


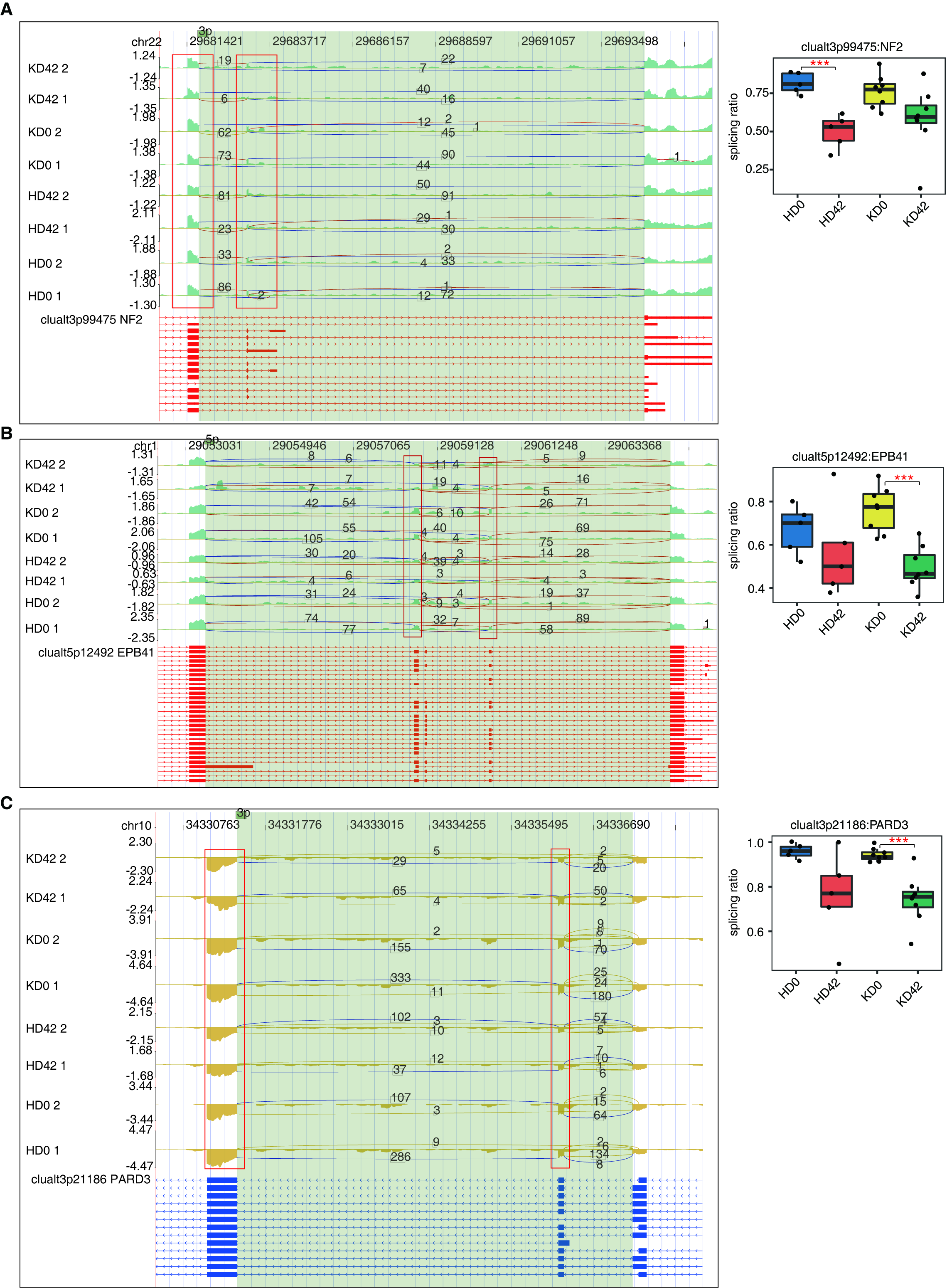


**Supplementary Figure 2.** Analysis of differential AS of wound healing-associated genes in skin tissue from KPI and HC. (A-C) The reads distribution shows clualt3p99475 NF2 and clualt5p12492 EPB41 and clualt3p21186 PARD3. Splice junctions were labeled with SJ reads the number, and the altered exon was marked out with a red box. The splicing events model is shown in the top panel. Boxplot in the bottom panel showing the splicing ratio profile of the splicing event. Boxplot showing splicing ratio of clualt3p99475 NF2 and clualt5p12492 EPB41 and clualt3p21186 PARD3 on the right.*p ≤ 0.05, **p ≤ 0.01,***p ≤ 0.001.


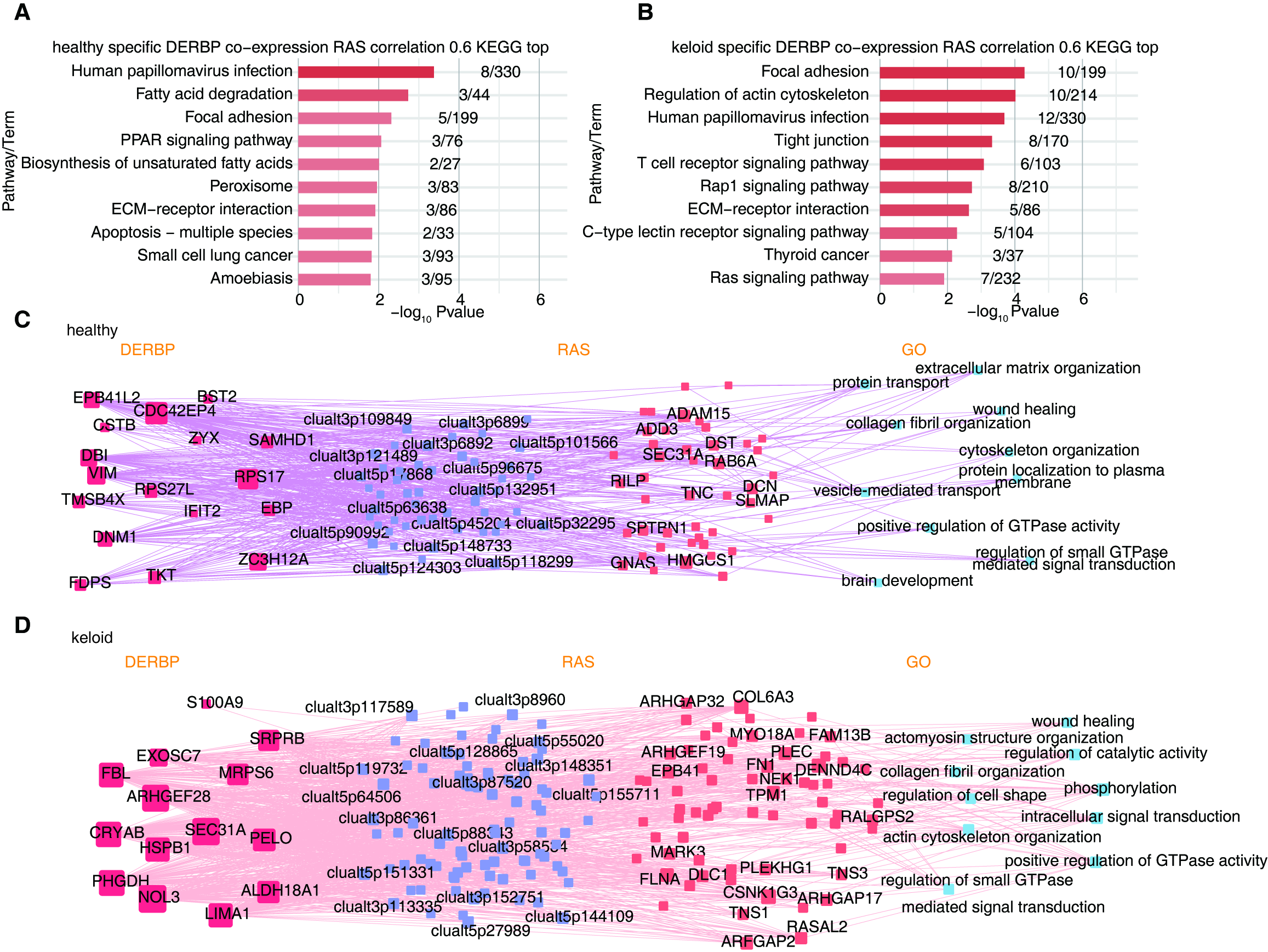


**Supplementary Figure 3.** Differentially expressed RBPs potentially affect AS of wound healing-associated genes in KPI and HC. (A) Bar plot showing the most enriched KEGG pathways results of RAS co-expressed by specific DERBP in HC. Cutoffs of p ≤ 0.01 and Pearson coefficient ≥ 0.6 or ≤ -0.6 were applied to identify the co-expression pairs. (B) Bar plot showing the most enriched KEGG pathways results of RAS co-expressed by specific DERBP in KPI. Cutoffs of p ≤ 0.01 and Pearson coefficient ≥ 0.6 or ≤ -0.6 were applied to identify the co-expression pairs. (C) Network diagram showing the top 10 GO terms (biological process) of RAS co-expressed by specific DERBP in HC. (D) Network diagram showing the top 10 GO terms (biological process) of RAS co-expressed by specific DERBP in HC.
